# Organs in orbit: how tissue chip technology benefits from microgravity, a perspective

**DOI:** 10.3389/frlct.2024.1356688

**Published:** 2024-03-07

**Authors:** Aditi Jogdand, Maxwell Landolina, Yupeng Chen

**Affiliations:** Department of Biomedical Engineering, University of Connecticut, Storrs, CT, United States

**Keywords:** tissue chip, microgravity, biomimetic, spheroid, mechanotransduction, stem cell

## Abstract

Tissue chips have become one of the most potent research tools in the biomedical field. In contrast to conventional research methods, such as 2D cell culture and animal models, tissue chips more directly represent human physiological systems. This allows researchers to study therapeutic outcomes to a high degree of similarity to actual human subjects. Additionally, as rocket technology has advanced and become more accessible, researchers are using the unique properties offered by microgravity to meet specific challenges of modeling tissues on Earth; these include large organoids with sophisticated structures and models to better study aging and disease. This perspective explores the manufacturing and research applications of microgravity tissue chip technology, specifically investigating the musculoskeletal, cardiovascular, and nervous systems.

## 1 Introduction

Researchers have begun to transition from traditional biomedical research methods to tissue chips to better understand complexities of the human body. While conventional monolayer cell culture and animal models are effective in the initial study of a condition or cure, more intricate processes are required to truly predict how these factors will perform. Tissue chips house cells akin to their natural state, implementing three-dimensional (3D) culture, semipermeable membranes, and mechanical stimulation to precisely model their typical environment (Yau et al., 2021; Rice et al., 2022). This allows researchers to accurately mimic the body and its pathologies, to better understand both diseases and possible therapeutics (Low and Tagle, 2016). These chips can incorporate microfluidic environments as well as controllable software that alters fluid flow and pressure to meticulously recreate the desired tissue (Bi et al., 2006; Yau et al., 2023a; Yau et al., 2023b).

In the past century, space exploration has greatly advanced at a low cost, as reusable spacecrafts and rocket performance improve (Mu et al., 2022a). Accordingly, microgravity has become much more accessible.

Microgravity simulators do exist on Earth. Experimental platforms such as 2D clinostats, rotating wall vessels, and random positioning machines attempt to reproduce the effects observed in true microgravity (Herranz et al., 2013). Interpreting data from these simulators can at times prove complex and erroneous (Ferranti et al., 2020a; Silvani et al., 2022) Another alternative are aircrafts that fly in parabolic maneuvers, creating true zero gravity conditions, albeit for less than 30 s at a time (Zheng et al., 2017; Shelhamer, 2016; Wang et al., 2015). However, these on-Earth simulations greatly reduce the cost and time associated with microgravity studies in orbit. Overall, these simulated microgravity conditions appear to recreate similar conditions to what’s experienced in space (Wuest et al., 2015). More studies are necessary to identify possible differences in true and simulated microgravity.

This perspective addresses the advantages and limitations of microgravityand their impact on the ability of models to properly mimic *in-vivo* conditions. Current research on the musculoskeletal, cardiovascular, and nervous systems is discussed and insights are provided on how implementation in microgravity could provide improved results.

## 2 Unique properties of space

A lack of gravity causes cells to rapidly adapt, impacting a variety of characteristics; *in-vitro* tissues form more sophisticated and organized structures, similar to those seen *in-vivo* (Chang and Hughes-Fulford, 2009). 3D cell aggregates, known as spheroids, thus may benefit from utilization in space. Spheroids mimic the natural spatial architecture of certain tissues, increasing cell-cell interactions and the intricacy of structures compared to conventional 2D cultures (Juarez-Moreno et al., 2022; Kadletz et al., 2015). While spheroids have been proven as effective models, they are not simple to produce. To alleviate these difficulties, some have turned to microgravity. Various cells exposed to microgravity saw an increase in cellular aggregation, passively improving the formation of both tissue and tumor spheroids (Masiello et al., 2014; Grimm et al., 2018; Aleshcheva et al., 2016; Buken et al., 2019).

Microgravity expedites both the fabrication time and the modeled disease progression of tissue chips. One cause is the altered differentiation and proliferation of stem cells in microgravity. Human induced pluripotent stem cells cultured on the International Space Station (ISS) for 6 weeks showed a modified expression of 2,000 genes compared to their Earth-cultured counterparts (Giulianotti and Low, 2019). This modified differences can prove beneficial for researchers; Hagiwara et al. found that endothelial progenitor cells exposed to microgravity show greater angiogenic potential, conceivably quickening the vascularization of future tissue chips (Hagiwara et al., 2018). Various other groups observed that mesenchymal stem cells are guided towards an osteogenic phenotype in space without the need for cytokines, accelerating the fabrication of bone tissue chips (Gambacurta et al., 2019; Cazzaniga et al., 2016) Cardiac tissue modeling has also shown potential; in addition to enhanced angiogenesis, the proliferation and differentiation of cardiomyocytes has been observed in microgravity (Jha et al., 2016). Similarly, microgravity bolsters proliferation, survival, and shortened cell cycles in neural crest stem cells (Han et al., 2021) while stimulating neuroprotective effects in mesenchymal stem cells (Otsuka et al., 2018).

Microgravity additionally eliminates loading force. In orbit, astronauts lose cartilage, muscle, and bone mass due to the drastic decrease in mechanical stimuli (Ganse et al., 2022; Comfort et al., 2021; Mu et al., 2022a; Grimm et al., 2016; Willey et al., 2011). For instance, bone cells are mechanosensory, sensing and relaying mechanical signals. During load-induced strain, these cells release signaling molecules (Wang et al., 2021), inducing either formation or resorption (Juhl et al., 2021; Man et al., 2022). Without sufficient loading, bone tissue will be catabolized (NIAMS, 2017). These effects, along with the others listed, are summarized in Table 1:

While these loading-related diseases can be studied on Earth, the methods to cause these catabolic effects in models are typically limited to inducing the downstream chemical signaling seen in mechanotransduction. However, many of these signaling pathways are not well understood. A single stimulus may cause the release of a multitude of factors. Even then, the dose and frequency of downstream signaling molecules must be greatly scrutinized to determine their similarity to real disease conditions. Microgravity, however, offers the ability to directly alter the physical stimulus that causes these downstream effects. Instead of comprehensively mapping each pathway, one can directly cause the lack of loading that induces this broad signaling naturally. Thus, microgravity provides a rigorous testing environment for therapies designed to fight against these conditions, offering a streamlined ability to evaluate possible therapeutics.

To take full advantage of these benefits, tissue chips must be optimally designed prior to their journey into LEO. Due to the limited amount of current research done in space, anticipating design issues, and correcting them when they occur, is a challenging endeavor. Computer-aided design (CAD) has the potential to steer clear of these limitations; CAD allows researchers to increase the intricacies of their chip design, perform extremely accurate measurements, and diversify methods of fabrication (Tsur, 2020). These factors could be utilized to develop improved tissue models for microgravity-based research before they even have to get in a rocket. In a similar vein, machine learning models can also be trained to review each study, developing new and improved methods to fine tune previous ideas. As more information is gathered, more will be understood by these models, resulting in continuously optimized tissue chip designs for use in microgravity.

In addition to its plentiful biological applications, researchers can also take advantage of microgravity’s physical properties to improve the fabrication of tissue chips. Due to the lack of gravity, forces such as convection and sedimentation do not occur in LEO. This allows polymeric tissue scaffolds formed in solution, such as hydrogels, to develop a much more homogeneous structure. This enhanced architecture should, in theory, promote higher levels of cell adhesion and interaction.

## 3 Advantages of microgravity

This review will focus primarily on how space may improve musculoskeletal, cardiovascular, and nervous system tissue chips. As of now, models of these systems can take the most advantage of microgravity.

### 3.1 Musculoskeletal system

The musculoskeletal system is crucial to support our body and preserve mobility. It comprises bone, muscle, cartilage, along with other specialized connective tissues. Musculoskeletal diseases, like osteoporosis and osteoarthritis, can be especially burdensome, as they directly impact one’s ability to be active.

Bone is a dynamic and complex tissue, constantly adapting to external mechanical stimuli; moderate loading will strengthen bone, but inadequate loading will diminish it (Rowe et al., 2022). To study this phenomenon, Paek et al. developed an effective high-throughput bone-on-a-chip platform to mimic the structure of an osteon, a functional unit of bone, to act as a drug screening platform for osteoporosis (Paek et al., 2023). The presence of sclerostin, in which causes bone resorption, was used to assess drug efficacy. While the therapeutic did decrease sclerostin concentration, the chip itself did not truly model a diseased state. Thus, it is unclear if the drug would be therapeutically effective. If taken to space, microgravity could recapitulate the onset of osteoporosis; additionally, the physical properties could generate a more homogeneous osteon structure. Similarly, Galvan et al. developed a tissue chip to model partially developed bone tissue (Galván-Chacón et al., 2022). The passive osteogenic differentiation and increased cellular arrangement could vastly improve their model. These theoretical approaches have been verified by animal models in LEO. Coulombe et al. confirmed the acceleration of osteoporosis in space, even observing the intricacies of osteoporotic development, like varying levels of bone loss depending on the skeletal maturity of the animal (Coulombe et al., 2021).

Chondral breakdown in LEO has also been observed in mice (Kwok et al., 2021). Some scientists have already taken advantage of this with novel tissue models. Grodzinsky et al. utilized microgravity to study inflammation-related interactions in a cartilage-bone-synovium model (Dwivedi et al., 2022; Low and Giulianotti, 2019a). Shi et al. and van Loo et al., may benefit from the influenced cell aggregation in LEO to further enhance their spheroid cultures to study hypoxic chondrocytes and overall cartilage formation, respectively (Shi et al., 2015; van Loo et al., 2024).

**FIGURE 1:**
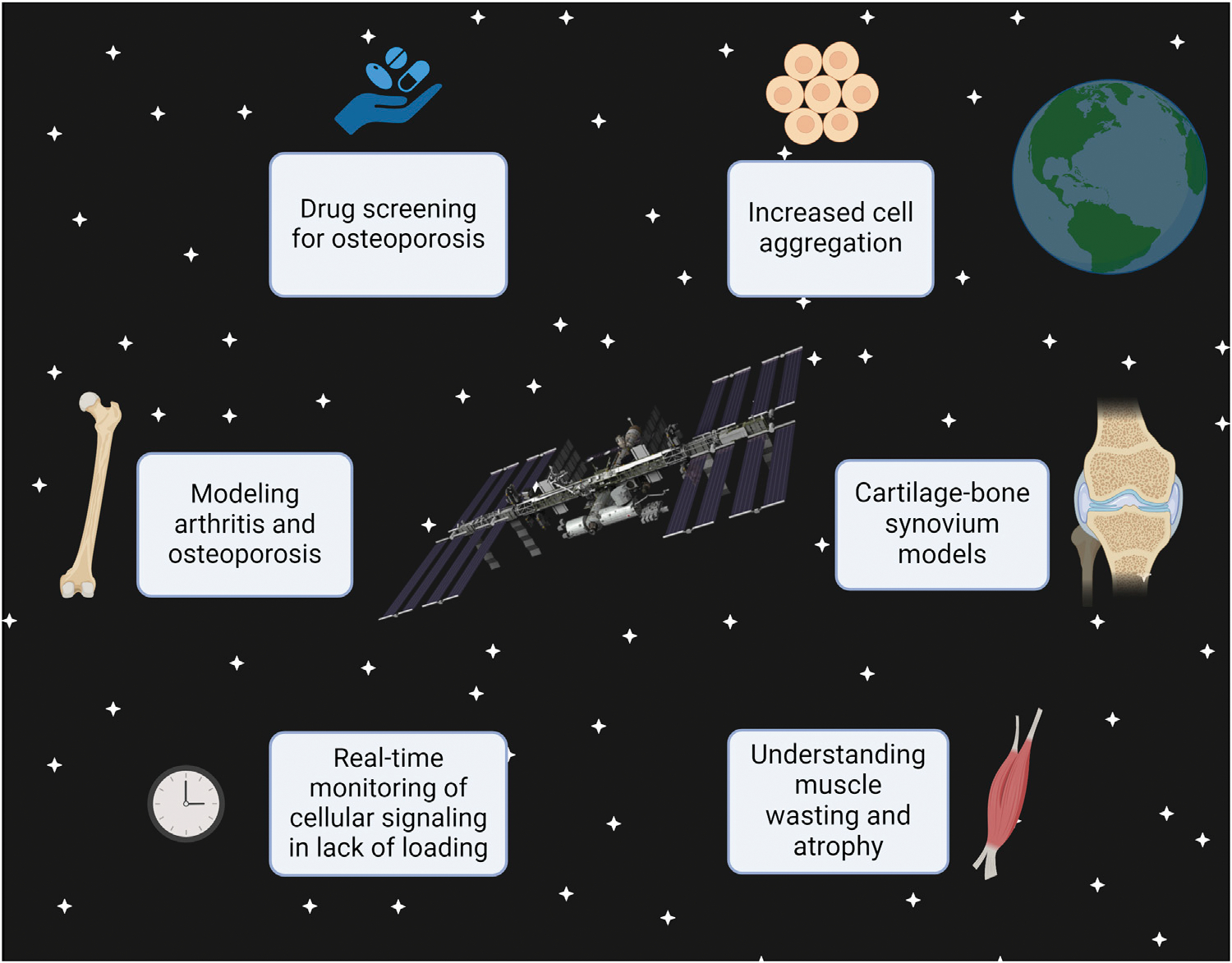
This figure demonstrates that future developments, such as using the 3D printer on the ISS, can allow scientists to manufacture tissue chips in space rather than Earth. The types of research that can be done in microgravity conditions are displayed, specifically using the musculoskeletal system as an example. This figure was created with Biorender.com.

Skeletal muscle has similar potential in microgravity. On Earth, Ortega et al. designed a muscle-on-a-chip to monitor two factors lead to muscle atrophy (Ortega et al., 2019). Muscle atrophy is a common consequence of aging (Altun et al., 2010), resulting from immobilization and disuse (Bodine, 2013). By employing Ortega et al.’s tissue chip in microgravity, scientists could observe how a lack of loading affects cellular signaling in muscle tissue, with real-time monitoring. This would eradicate the current need for ethically ambiguous animal models while providing invaluable information that even on-Earth tissue models may implement to accurately mimic atrophic progression. The effects of microgravity on skeletal muscle atrophy have already been well documented in tissue chips taken to the ISS (Parafati et al., 2023), astronauts in orbit (Lee et al., 2022), and LEO-based animal models (Okada et al., 2021). Interestingly, these effects remain with cells after their return to Earth (Takahashi et al., 2021). These models, along with other musculoskeletal chips, have potential to see the improvements outlined in Figure 1:

### 3.2 Cardiovascular system

Cardiovascular diseases are a leading cause of mortality, affecting over 17 million people each year (Amini et al., 2021). As microgravity has promoted angiogenesis (Morbidelli et al., 2021; Shi et al., 2017), induced spheroid formation in endothelial cells (Dittrich et al., 2018), and expedited the differentiation of cardiac progenitors from pluripotent stem cells (Jha et al., 2016), it may provide an excellent environment for cardiac tissue modeling.

Figtree et al. (2017) cocultured cardiac myocytes, endothelial cells, and fibroblasts to study cardiac fibrosis in vascularized cardiac spheroids. While their model was successful, the increased cellular aggregation and angiogenic potential in microgravity would likely further strengthen their model. Additionally, adapting successful animal cardiac models (Liu et al., 2021; Chen et al., 2019) to microgravity-exposed tissue chips might provide a more repeatable, accurate method to study drug efficacy, especially as microgravity could accelerate disease progression (Lynch et al., 2007; Walls et al., 2020).

Vascular damage is another factor of cardiovascular disease that has been observed during zero gravity conditions. To understand these outcomes, large scale artery dynamics were modeled in simulated microgravity, providing key insights on their modified mechanics (Caddy et al., 2024).

Tang et al. created a heart-on-a-chip platform with induced pluripotent stem cells to evaluating therapeutics and study the importance of the endothelial layer. The aforementioned impacts on cell differentiation and organization observed in microgravity could assist this model even further in its accuracy (Tang et al., 2022). Collectively, cardiovascular tissue chips have not yet implemented microgravity as much as musculoskeletal models, yet they maintain a high degree of potential.

### 3.3 Nervous system

The nervous system grants us the ability to breathe, think, and move, making it uniquely devastating when this essential network fails. In 2019, 10 million died due to neurological disorders, with a burden of disease that continues to increase (Ding et al., 2022; Feigin et al., 2020). Thus, understanding its underlying processes are essential. To perform these investigations, tissue chips are employed as *in-vitro* blood-brain-barrier (BBB) models to develop permeable therapeutics and recreate malignant tumors.

Neurodegenerative diseases such as Alzheimer’s disease (AD), Parkinson’s disease (PD), and multiple sclerosis affect millions. These chronic conditions devastate both patients and their families (Praznikov Victor, 2022). Tissue chips offer an opportunity to better grasp the complexities of these diseases. Park et al. developed a microfluidic chip with 3D neurospheroids to precisely capture *in-vivo* conditions seen in AD, such as reduced viability and disrupted neural networks (Grimm et al., 2022). By promoting increased spheroid formation in microgravity (Grimm et al., 2020), this already effective model could be further perfected to model AD. Shin et al. similarly created a chip to model AD-associated BBB dysfunction, demonstrating how plaque deposition increases BBB permeability (Shin et al., 2019). In LEO, this model could experience increased cell-cell interaction, cell adhesion, and possible accelerated disease progression (Low and Giulianotti, 2019b; Yau et al., 2023b) to further improve their AD tissue chip design. The study of the BBB under microgravity has already been a topic of interest for scientists. In fact, Hinojosa et al. have sent their model of the BBB to the ISS to studying real-time cell-cell interactions (Reporter, 2020). In 2018, Leong et al. developed a microfluidic device for use in space to understand the relationship between the brain and vasculature, as well as the impact of neuropsychiatric drugs (Reporter, 2022).

Tsybko et al. studied how glial cell-derived neurotrophic factor (GDNF), which can promote the recovery of motor function in PD (Björklund et al., 1997), is affected in mouse brains exposed to simulated microgravity (Tsybko et al., 2015). This study also found that spaceflight had negative effects on the dopamine system, with dysregulation of genetic control over GDNF being a potential cause. This offers new insight to protect the health of astronauts, as well as new methods to create Parkinsons-on-a-chip models by using microgravity.

In addition to AD and PD, brain tumor models may benefit from microgravity. Glioblastomas are aggressive tumors with low life expectancies (Jovčevska, 2020). This level of importance makes them prime candidates for accelerated study in microgravity. To better model BBB permeability observed in glioblastomas, Silvani et al. used microgravity to decrease the expression of tight junction proteins, directly increasing permeability. This low gravity-induced permeability could also act therapeutically, temporarily allowing chemotherapeutics to cross the BBB (Silvani et al., 2021). Other brain tumor models are likely to benefit from the enhanced tumor formation and structural architecture seen in microgravity (Samiei et al., 2020).

Similar to Silvani et al.’s observations, microgravity has been seen to decrease the BBB’s tight junction proteins in mice. This acceleration of neurodegeneration is another concern for astronauts. A novel BBB-on-a-chip could be created to visualize how microgravity affects the brain via specific gene expression (Yan et al., 2021) and oxidative stress (Chen et al., 2009) previously observed in animal studies. Understanding the mechanism behind these spaceflight-induced physiological changes can allow scientists to develop possible solutions.

### 3.4 Physiological effects of microgravity

While microgravity has its benefits for furthering research, astronauts who spend significant amounts of time in LEO observe changes in their tissue functions. In this vein, tissue chips can be utilized to understand how prolonged exposure to microgravity impacts systems such as the musculoskeletal, cardiovascular, and nervous systems are impacted.

The aforementioned loss of musculoskeletal tissue likely arises from a loss of load, but it may be exacerbated by radiation (Hamilton et al., 2006).

This radiation has also been observed to increase oxidative stress and inflammation in the carotid arteries of astronauts (Lee et al., 2020) along with structural remodeling and fibrotic alterations of cardiac tissue (Xu et al., 2021). Tissue chips may be the ideal option to study these phenomena. This research may also prove beneficial on Earth, providing insights into the heart damage seen in thoracic cancer radiation therapy (Schaue and McBride, 2015).

Venous thrombosis (Auñón-Chancellor et al., 2020), anemia, and decreased hemoglobin concentration (Trudel et al., 2020; Nicogossian, 2003) have all been observed in astronauts on the ISS. Further haemodynamic studies, such as Caddy et al.’s, are necessary to resolve these issues (Caddy et al., 2024).Alternatively, the hemoglobin degradation and hemolysis (Trudel et al., 2022) can be utilized in tissue chips to evaluate mechanisms of hemolysis-causing diseases, such as malaria, or test possible therapies. Anemia also increases the susceptibility to infectious diseases like tuberculosis (Gelaw et al., 2021), providing a more rigorous testing apparatus if implemented.

Gray and white matter alterations, cognitive and motor ability reductions, and the development of neuro-optic syndrome have all been detected after spaceflight (Riascos et al., 2019; Marfia et al., 2022; Roberts et al., 2019). The study of brain organoids in microgravity is necessary to understand the impact of radiation on astrocytes and neural architectures, like the BBB, to avoid these outcomes (Roggan et al., 2023).

### 3.5 Limitations of research in zero gravity

While microgravity has the potential to further biomedical research in a new frontier, it must be scrutinized before it can achieve clinical relevance. The same changes in gene expression that benefit researchers, such as cell differentiation and proliferation, can be problematic when trying to apply results from drug studies to human subjects. A drug may be effective in microgravity when cells express certain genes, but when that same drug is implemented on Earth, it may not completely translate its efficacy. Perhaps a gene needed for the drug’s success was upregulated in microgravity but downregulated on Earth. Additionally, microgravity causes complex changes cytoskeleton organization (Wu et al., 2022). Some studies have shown that microgravity decreases expression of actin while others found the opposite to occur (Bradbury et al., 2020).

These observations also depend on cell type; in human neuroblastoma cells, microgravity showed microtubule bending but had no influence on actin dynamics (Rösner et al., 2006). In glioma cells, microgravity resulted in B-tubulin disorganization (Wang et al., 2016). However, cardiovascular progenitor cells cultured in space increased the expression of cytoskeleton genes (Baio et al., 2018).

Space research has striking potential in terms of tissue chip research, but the in-space changes of cells and disease progression must be investigated further before it can be accepted as a true replica of disease conditions on Earth. It is possible that exposure to microgravity itself could alter a disease’s mechanisms rather than only accelerating their progression. Also, in-space facilities have strict loading capacity and experimental limitations, perhaps leading to oversimplification, and therefore inaccuracy, of the tissue chip models on board (Mu et al., 2022b; Ferranti et al., 2020b). In addition, cosmic radiation may greatly impact the efficacy of these tissue chips (Li et al., 2018; Nwanaji-Enwerem et al., 2022; Soucy et al., 2011; Belli et al., 2002). Perhaps protective apparati could be developed to mitigate the exposure to this radiation, or at the very least attenuate it to a lesser degree. In all, these observations obtained from microgravity-exposed tissue chips require more time before they can be directly utilized in clinically relevant comparisons. They can, however, provide a foundational understanding, whose breadth will continue to expand over time.

## 4 Discussion

As tissue chip and space travel technologies advance, scientists can fully utilize the accelerated disease progression, cell aggregation, spheroid formation, and variation in genetic expression in microgravity to further biomedical research. These characteristics will lead to a deeper understanding of the mechanisms, signaling, and therapeutic possibilities for an array of conditions. This has begun for the musculoskeletal, cardiovascular, and nervous systems, and others will likely follow suit. In the future, it is likely microgravity will become ubiquitous in this field, providing researchers with passive methods to most accurately model disease and rigorously assess therapies. Through this investigation of accelerated disease conditions and unique properties, microgravity can aid us in creating effective and long-lasting therapies, using space to improve the quality of life for us here on Earth.

## Figures and Tables

**TABLE 1:** This table showcases the multitude of effects that befall the human body in microgravity. The musculoskeletal system is exposed to a complete lack of mechanical stimuli, causing muscle atrophy, cartilage degradation, and bone resorption. Further, the blood brain barrier undergoes an increase in permeability, compromising the integrity of the brain’s primary protective system. Microgravity also accelerates tumor formation. In the heart, it provides modified mechanisms of blood flow in addition to hemoglobin degradation and hemolysis. Overall, these results are due to the altered gene expression, acceleration of disease, exposure to radiation, and exposure to the physical properties of microgravity in space.

Organ system	Purported/Observed effects of μg	Presumed causes
Nervous	↑ BBB permeability	Altered gene expression
	↑ Proliferation and survival of neural crest stem cells	
	Shortened cell cycles	
	↑ Neuroprotective effects	Acceleration of degenerative and aging-based diseases
	↑ Tumor formation	
Cardiovascular	Altered mechanics of blood flow	Exposure to increased levels of radiation
	↑ Angiogenesis	
	↑ Proliferation and differentiation of cardiomyocytes	
	Hemoglobin degradation	Exposure to unfamiliar physical properties of microgravity
	Hemolysis	
Musculoskeletal	Passive osteogenic differentiation	Lack of mechanical stimuli
	Bone resorption	
	Cartilage degradation	
	Muscular atrophy	

## Data Availability

The original contributions presented in the study are included in the article/supplementary material, further inquiries can be directed to the corresponding author.
